# p.Arg82Leu von Hippel-Lindau (*VHL*) Gene Mutation among Three Members of a Family with Familial Bilateral Pheochromocytoma in India: Molecular Analysis and *In Silico* Characterization

**DOI:** 10.1371/journal.pone.0061908

**Published:** 2013-04-23

**Authors:** Anulekha Mary John, George Priya Doss C, Andrew Ebenazer, Mandalam Subramaniam Seshadri, Aravindan Nair, Simon Rajaratnam, Rekha Pai

**Affiliations:** 1 Department of Endocrinology, Christian Medical College, Vellore, Tamil Nadu, India; 2 Medical Biotechnology Division, Centre for Nanobiotechnology, School of Biosciences and Technology, VIT University, Vellore, Tamil Nadu, India; 3 Department of Pathology, Christian Medical College, Vellore, Tamil Nadu, India; 4 Department of Endocrine Surgery, Christian Medical College, Vellore, Tamil Nadu, India; Scuola Superiore Sant’ Anna, Italy

## Abstract

Various missense mutations in the *VHL* gene have been reported among patients with familial bilateral pheochromocytoma. However, the p.Arg82Leu mutation in the *VHL* gene described here among patients with familial bilateral pheochromocytoma, has never been reported previously in a germline configuration. Interestingly, long-term follow-up of these patients indicated that the mutation might have had little impact on the normal function of the *VHL* gene, since all of them have remained asymptomatic. We further attempted to correlate this information with the results obtained by *in silico* analysis of this mutation using SIFT, PhD-SNP SVM profile, MutPred, PolyPhen2, and SNPs&GO prediction tools. To gain, new mechanistic insight into the structural effect, we mapped the mutation on to 3D structure (PDB ID 1LM8). Further, we analyzed the structural level changes in time scale level with respect to native and mutant protein complexes by using 12 ns molecular dynamics simulation method. Though these methods predict the mutation to have a pathogenic potential, it remains to be seen if these patients will eventually develop symptomatic disease.

## Introduction

Pheochromocytomas (PCC) are rare tumors arising from the chromaffin-cells of the adrenal medulla. They might arise sporadically or in association with any one of the cancer syndromes that includes von Hippel-Lindau (*VHL*) disease, neurofibromatosis type 1 (*NF 1*), multiple endocrine neoplasia type 2 (*MEN 2*) and mutations in any one of the mitochondrial succinate dehydrogenase complex (SDHx) genes [1], [2]. More recent data indicates that mutations in *SDHAF2* and *TMEM127* may also be associated with PCCs [3], [4]. Neumann et al have shown that ∼30% of unselected cases of apparently sporadic PCC carry germline mutations though the percentage may vary slightly between various studies. PCCs are rarely bilateral or multifocal [5], [6]. However, the presence of bilateral PCC especially among young patients increases their risk of carrying a mutation and emphasizes the need to screen such patients for all susceptibility genes associated with PCC [7].

von Hippel-Lindau (*VHL*) disease, described first in the late 1800s, is an autosomal dominant disorder that could increase susceptibility to a range of tumors, both benign and malignant, including retinal hemangioblastomas, renal cysts and renal cell carcinoma, PCCs and other tumors [8]. PCCs were described in detail as a syndrome associated with *VHL* disease only in 1964 [9], though a lot more is now known on the phenotype-genotype associations in *VHL* disease [8]. While PCC is not seen among patients with type 1 *VHL* disease, it is a common feature associated with type 2 disease. Type 2 disease is further subgrouped into 2A, 2B, 2C where type 2C is known to be associated with only PCC. Also the pattern of mutations in these subgroups appears to be distinct [10] where type 1 *VHL* disease is known to be associated with deletions and truncating mutations while type 2 disease is associated with missense point mutations, which in turn is believed to reflect the degree of functional change it can cause to the *VHL* gene product [8]. We describe the details of three members a family who presented with only bilateral pheochromocytoma, where *VHL* gene mutations were eventually detected when the testing system became available as part of a routine screening of patients with PCC. The presence of a novel mutation, never described before in germline configuration, among these patients, also prompted us to undertake a detailed *in silico* analysis of the mutation to determine its pathogenic/deleterious potential.

## Materials and Methods

### Patients

#### Patient 1

The index case was a 14 year-old boy who presented to us in 1971 with severe hypertension. On evaluation he was found to have a right adrenal mass with elevated urinary VMA (normal <7 mg/24 hr), confirming the diagnosis of a right adrenal pheochromocytoma. He underwent right adrenalectomy and the post-operative period was uneventful. He was subsequently lost to follow. In 1979, he presented to us again with severe headache and recurrent seizures. On examination his pulse rate was 100/min, blood pressure was 160/130 mm of Hg. Cardiac auscultation revealed a loud 2^nd^ heart sound and a 3^rd^ heart sound. There were no murmurs. Examination of the abdomen revealed a palpable mass in the left hypochondrium. This mass was confirmed as a pheochromocytoma. After adequately controlling his blood pressure he underwent left cortical sparing adrenalectomy. The post-operative period was uneventful. He was on steroid replacement that was subsequently withdrawn after his serum cortisol levels had normalized. His 8 AM cortisol after withdrawal of steroids was 12 µg/dl.

**Figure 1:**
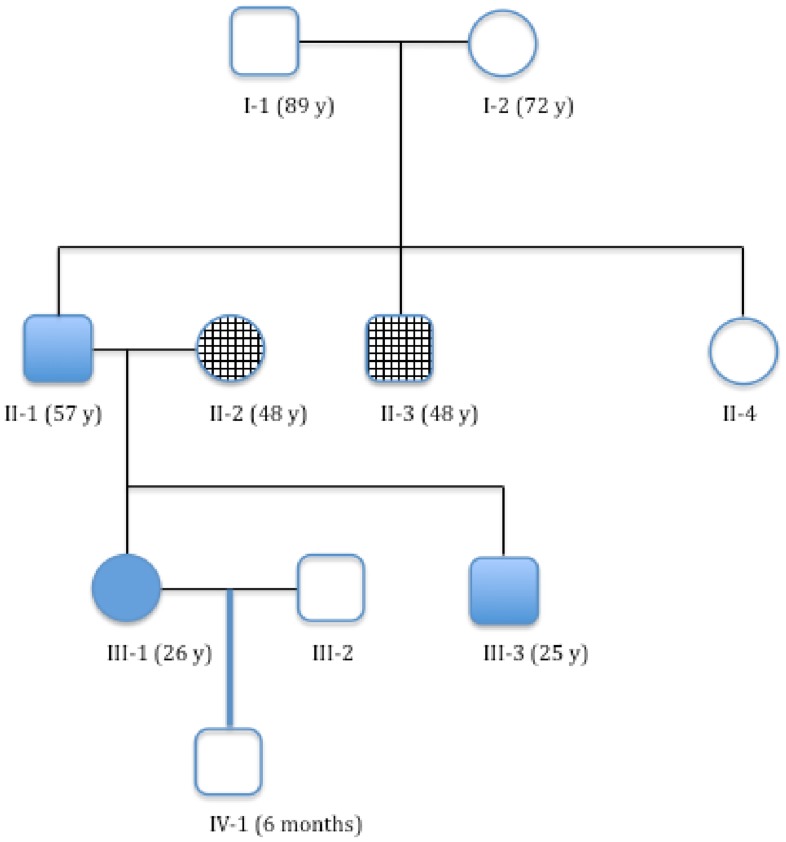
Current family tree (2012) showing the affected members; Each generation tested is marked with roman numeral; squares represent male and circles female; blank squares and circles represent members who were not tested; checkered were tested and found negative and blocked were positive for mutation.

He subsequently got married and had two children as shown in Figure 1, who also eventually developed similar tumors.

#### Patient 2

His eldest son presented to us at 10 years of age with severe hypertension. He was found to have bilateral pheochromocytoma. He underwent bilateral adrenalectomy in 1998. Subsequently he has remained asymptomatic.

#### Patient 3

**Figure 2:**
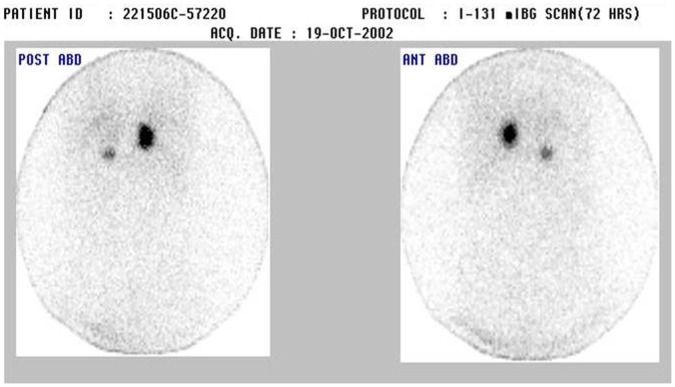
MIBG scan showing bilateral adrenal tumors.

**Figure 3:**
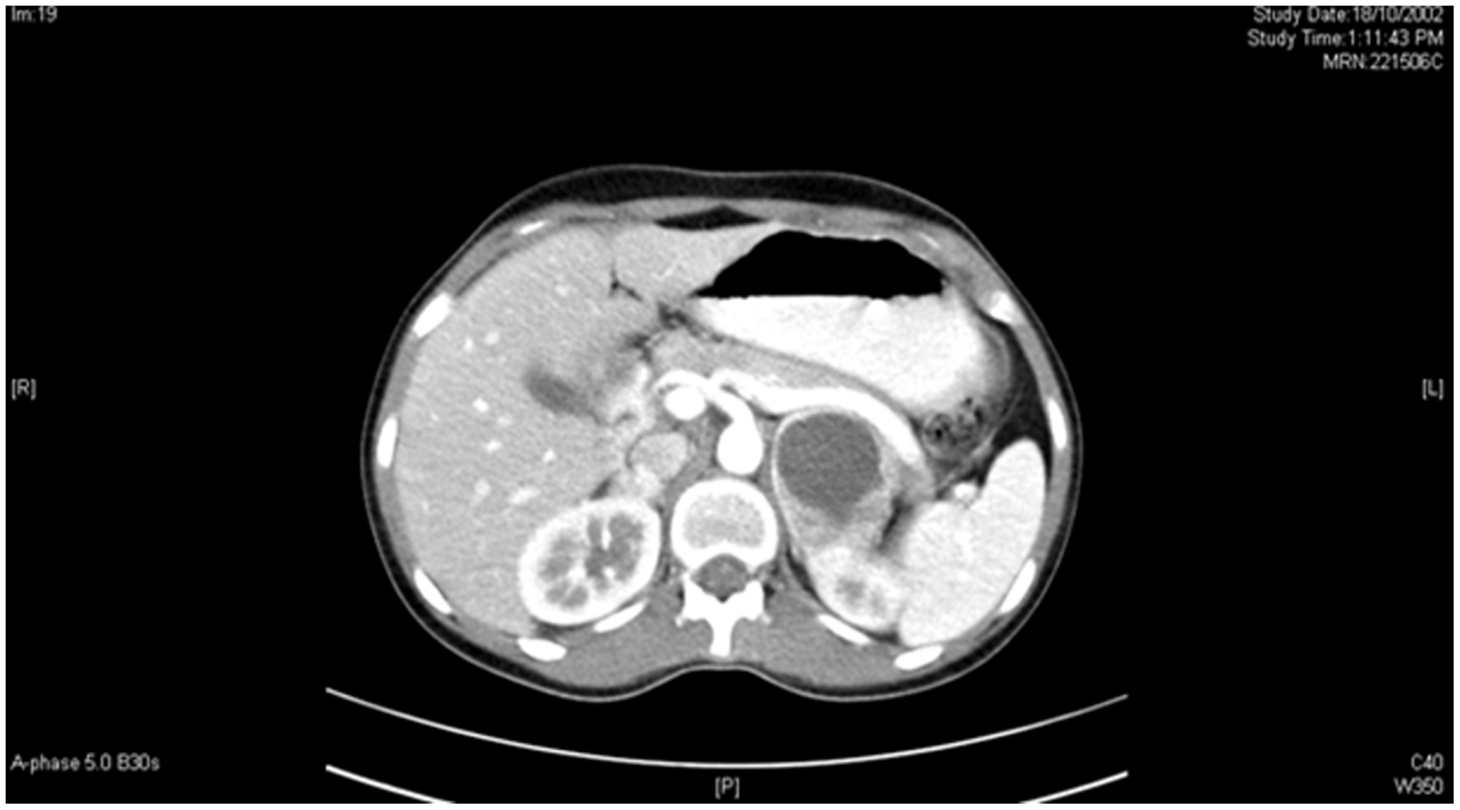
CT scan showing bilateral adrenal tumors.

His younger daughter was 17 years old when she presented to us in 2002, with palpitations, chest pain, and blurring of vision. On examination her heart rate was 100/min and blood pressure was 150/100 mmHg. Cardiovascular system examination revealed a loud 2^nd^ heart sound and a 3^rd^ heart sound. A short systolic murmur was heard along the left sternal border. She had bilateral papilledema with hemorrhages & exudates, and a macular star. There was no corneal nerve thickening or retinal angiomas. Rest of the systemic examination was normal. 24 hours urinary VMA on 2 occasions was elevated 8 and 8.1 mg (normal <7 mg/24 hr). MIBG scan (Figure 2) and CT scanning of the abdomen (Figure 3) confirmed the diagnosis of bilateral adrenal pheochromocytoma. She had a 2.5×2 cm right adrenal mass and a larger 5 4 cm adrenal mass on the left side. After adequate control of blood pressure she underwent bilateral adrenalectomy in 2002. The tumors were excised and the adrenal cortices were preserved on both sides. The post-operative period was uneventful. Biopsy confirmed the diagnosis of bilateral pheochromocytoma.

All the three cases were reviewed in 2012 and they have remained asymptomatic. Case 3 is now married and has a 6 months old baby. The pregnancy was uneventful.

#### Mutational analysis

**Figure 4:**
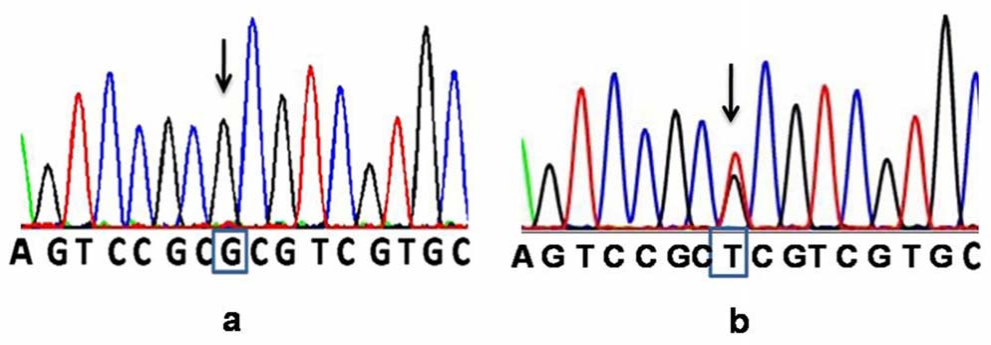
Electropherograms showing a wild type and the p.Arg82Leu mutation in the index patient.

The patients included in this manuscript were enrolled as part of a study on pheochromocytoma that was approved by the institutional review board of the Christian Medical College, Vellore (min no. 6869 dt 29/07/2009). The study commenced in 2010 when genetic analysis for *VHL* gene became available at our institution. All three members of this family with history of PCC were by then >18 years and written informed consent was obtained from each member. Two millilitres of peripheral venous blood was collected from each case. Two hundred microlitres was used for extraction of genomic DNA using QIAamp DNA blood minikit (QIAGEN, Hilden Germany). Twenty picomoles of primers were used for amplification of the VHL exons 1, 2 and 3 using previously published primers [11]. The reaction was carried out in a 25 µl volume containing 1 U of amplitaq gold (Applied Biosystems, USA) and amplification was carried out in a Veriti thermal cycler (Applied Biosystems, USA). The PCR products were detected using a 1.5% agarose gel and both the sense and antisense strands for all 3 exons were sequenced using the ABI PRISM 3130 genetic analyzer with the ABI PRISM BigDye Terminator Cycle Sequencing Ready Reaction Kit (Applied Biosystems, USA). The sequences were compared to the wild type sequence and both www.sangers.ac.in and www.lovd.com databases were checked to verify if the mutation (Figure 4) was previously reported.

#### In silico analysis

*In silico* analysis is a widely used approach for the prediction of unclassified variants as pathogenic or benign based on prediction scores. Most of the *in silico* tools utilize sequence, structure information along with physical properties of amino acids to calculate the prior probability of pathogenicity for unclassified variants. Six widely used mutation pathogenicity prediction tools were used for analysis in this study. Evolutionary information based SIFT [12], I-mutant3 SVM profile [13], PhD-SNP SVM Profile [13] were used; for a combination of protein structural and/or functional parameters and multiple sequence alignment derived information the tools included MutPred [14], PolyPhen2 [15], and SNPs & GO [16]. The machine learning methods utilize random forests (RF) (MutPred) or support vector machines (SVMs) (PhD-SNP, SNPs & GO, I-mutant3) for classification, while all the other methods classify variants according to Bayesian methods (PolyPhen2), or mathematical operations (SIFT). Position specific conservation pattern of p.Arg82Leu in VHL protein was analyzed using an empirical bayesian inference ConSurf server [17]. The conservation scores calculated by ConSurf are a relative measure of evolutionary conservation at each sequence site of the target chain. Lastly, we applied an automatic mutant analysis web server, Project Have yOur Protein Explained (HOPE) to analyze the effect of a certain mutation on the protein structure [18].

#### Molecular dynamics simulation procedure

The potential impact of the mutation p.Arg82Leu was evaluated by visual inspection of environment of the altered residue in the crystal structure of the VHL protein. The structure of the VHL protein (PDB ID 1LM8) contains two primary functional domains: the α-domain (155–192 aa), and the β-domain (63–154 and 193–204 aa) [19]. p.Arg82Leu is located in the functional β-domain of the VHL protein. For molecular dynamics study, native and mutant structures were generated based on the crystal structure (PDB ID 1LM8) of VHL protein by using Swiss PDB Viewer [20]. MD simulations for native and mutant models were done with MD simulation package GROMACS 4.0.5 that adopts GROMOS96 43a1 force field parameter for energy minimizations. Energy minimized structures of the native and mutant structure were used as a starting point for MD simulations. Both the structures were solvated in a cubic box with wall extending at least 0.9 nm from all atoms, and filled with SPC [21] water molecules. A periodic boundary condition was applied that the number of particles, pressure and the temperature was kept constant in the system. In order to obtain electrically neutralized system, we utilized GENION procedure from the GROMACS package to replace random water molecule with Na^+^ or Cl^–^ ions. The temperature was kept constant by using a Berendsen algorithm [22] with a coupling time of 0.2. The minimized system was equilibrated for 10000 ps each at 300 K by position restrained molecular dynamics simulation in order to soak the macromolecules into the water molecules. The equilibrated systems were then subjected to molecular dynamics simulations for 12 ns each at 300 K. In both the simulations temperature was kept constant at 300 K. The particle mesh Ewald method [23] was used to treat long-range coulombic interactions and the simulations performed using the SANDER module [24]. The SHAKE algorithm was used to constrain bond lengths involving hydrogen’s permitting a time step of 2 fs and coordinates were saved at regular time intervals of every 1 ps. The Van der Waals force was maintained at 1.4 nm, and coulomb interactions were truncated at 0.9 nm.

#### Analysis of molecular dynamics trajectories

Structural properties of the native and mutant model of VHL protein were calculated from the trajectory files with the built-in functions of GROMACS 4.5.3. The trajectory files were analyzed through the use of g_rmsd and g_rmsf GROMACS utilities in order to obtain the root-mean-square deviation (RMSD) and root-mean square fluctuation (RMSF) values. Number of distinct hydrogen bonds formed in protein during the simulation was calculated using g_hbond utility. Number of hydrogen bond determined on the basis of donor-acceptor distance smaller than 3.9 nm and of donor–hydrogen-acceptor angle larger than 90 nm [25]. Salt bridge formed in VHL protein was analyzed by using g_salt GROMACS. If the distance is ≤4.0 nm the pair is counted as a salt bridge [26]. Radius of gyration was analyzed by the use of gyrate. Further solvent accessible surface area calculated by g_sas utility. In order to generate the three-dimensional backbone RMSD, RMSF of carbon-alpha, hydrogen bond, salt bridge, backbone gyration and SASA analysis and motion projection of the protein in phase space of the system were plotted for both the simulations using Graphing, Advanced Computation and Exploration (GRACE) program.

## Results and Discussion

This is the first report of a germline p.Arg82Leu (c.245 G>T) missense mutation being reported among three patients from a family with bilateral pheochromocytoma (Fig. 4). This mutation has however, been reported previously as a somatic mutation seen among pheochromocytomas/paragangliomas characterized by BAC array comparative genomic hybridization and somatic mutation screening by Burnichon N *et al* [27]. Also, two other reports have described a mutation at the same codon, though in both the cases the change in amino acid was from argnine to proline (p.Arg82Pro) [28], [29]. Most cases affected by the p.Arg82Pro mutation interestingly have been documented to develop retinal hemangioblastomas indicating the pathogenicity of this amino acid change. However this is in contrast to the present study, where the mutation does not appear to have affected the VHL protein entirely, as evinced by the absence of progression of the disease and absence of appearance of other features that are characteristic of VHL disease. A similar observation on the absence of overt VHL disease has been documented in another study on VHL mutation associated PCCs [30]. It is possible that the altered protein retains its wild type ability to bind to the VEC complex protein and as suggested previously by Clifford et al [31], the phenotype of VHL disease (type 2C) associated with only PCC perhaps truly retains its ability to regulate the transcription factors of HIF normally. Stebbins et al provide further evidence that in type II VHL disease associated with only pheochromocytoma there is a strong bias against hydrophobic core mutations resulting in a protein that is mostly associated with local defects i.e. mild loss of function (19). Further, Ong et al [32] have also noted that missense mutations that code for amino acids on the surface of the VHL protein are more likely to be associated with higher rates of PCC. These points taken along with the fact that all three patients in this report presented only with bilateral PCC and continue to be asymptomatic on long follow-up suggests that p.Arg82Leu mutation reported here is plausibly, a mutation that is associated predominantly and exclusively only with PCC. However, the role played by this mutation in the development of PCC itself, would be more difficult to deduce. Though the VHL protein is known to play a role in fibronectin extracellular matrix assembly and the role of two other mutations viz., L188V and V84L have been investigated [33], in this context the absence of experimental proof makes it purely speculative to believe that p.Arg82Leu could have played a similar role in the development of PCC.

Patients with PCC normally present during their 3^rd^ or 4^th^ decade of life.^3^ However, the mean age of diagnosis of PCC in VHL disease is much lower at ∼20 years with a clear predisposition to an early onset of tumors [34]. However, a recent report shows that pheochromocytoma in VHL disease could develop as early as 2 years of age [35]. The mean age at presentation of pheochromocytoma among patients in this report was 16.3 yrs. Interestingly, one of the two off-springs presented with PCC earlier (10 years) than the index case (14 years) concurring with other studies which have reported the onset of tumors to be earlier in the subsequent generations. However, it is now fairly well established that the risk of developing pheochromocytoma in these families could largely depend on the type of germline mutation in the *VHL* gene [8].

The *VHL* gene, identified in 1993, is located on chromosome 3p25–26. It is a tumor suppressor gene that encodes a protein that regulates hypoxia-induced proteins [7]. Like in many other conditions, VHL syndrome is also explained by a ‘two hit model’ [8]. There is a germ line mutation (first hit) that inactivates one copy of the *VHL* gene in all cells. For VHL-associated tumors to develop there must be loss of expression of the second normal allele (second hit), through either somatic mutation or deletion of the second allele, or through hypermethylation of its promoter. More than 500 germ line mutations have been identified to date [32] and the mutational pattern associated with the types of VHL disease is also fairly well characterized [36], [37]. Most mutations associated with type 2 VHL syndrome are known to be missense mutations while deletions and truncation are often associated with type 1 VHL syndrome. Further, the degree of functional change in *VHL* gene product is believed to differ with different VHL mutations and mutations that do not cause a total loss of function but are associated with a risk of pheochromocytoma, have been described previously [37], [38], [39], similar to the p.Arg82Leu mutation reported here. The authors have therefore also made an attempt to characterize this mutation further by an *in silico* approach, to determine its potential in being deleterious/pathogenic. Different working groups have applied various *in silico* methods as an integrated model to assess the pathogenicity of unclassified variants [40], [41], [42], [43], [44], [45] and have gradually gained acceptance in characterizing mutations.

### Identification of Deleterious Variants in *VHL* Gene by Using *in silico* Tools

**Figure 5:**
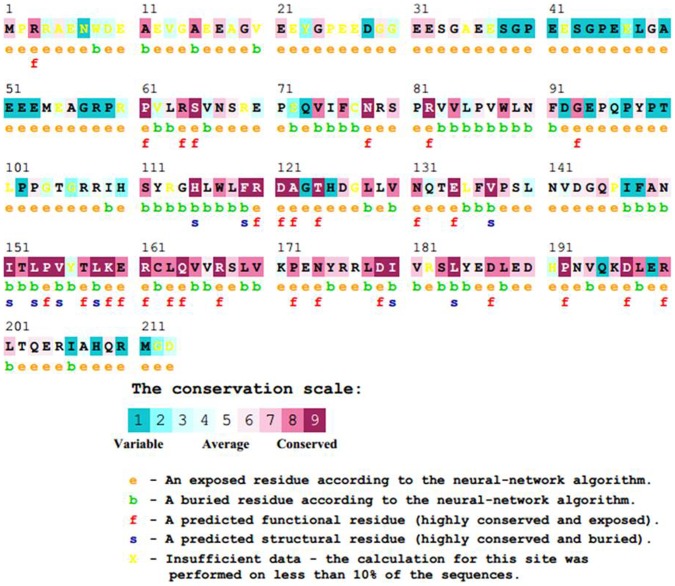
The conservation pattern of amino acid sequence in VHL protein by ConSurf. The location of amino acid residues in VHL protein based on the evolutionary conservation pattern. Color intensity increases with degree of conservation (Color figure online).

Identifying variations and determining the pathogenic potential of single nucleotide polymorphisms (SNPs) responsible for specific phenotypes is a difficult task in the laboratory. However, the availability of sophisticated computational tools that efficiently discriminate the pathogenic variants from benign in a fast and accurate manner has changed the scene phenomenally. Here, we analyzed VHL variant p.Arg82Leu with six different *in silico* tools namely SIFT, I-Mutant3, PhD-SNP, MutPred, PolyPhen2 and SNPs&GO in order to determine the pathogenic potential of the variant. Table 1 shows the prediction scores of all the six tools for the variant p.Arg82Leu of VHL protein. Each tool adopts different strategies for the classification of variant. SIFT makes inferences from sequence similarity using mathematical operations by constructing Multiple Sequence Alignments (MSA) and considers the position of the missense variant. Further, based on the amino acids appearing at each position in the MSA, SIFT calculates the probability, that a missense variant is tolerated. SIFT predicted that the variant p.Arg82Leu is deleterious with score 0.04. I-Mutant-3, a support vector machine starting from the protein sequence or structure discriminates between stabilizing, destabilizing and neutral mutations. I- mutant-3 predicted that the variant p.Arg82Leu is related to the disease state. PhD-SNP is a prediction method based on single-sequence and sequence profile based support vector machines trained on Swiss-Prot variants [46]. Single-sequence SVM (SVM Sequence) classified the missense variant to be pathogenic or neutral based on the nature of the substitution and properties of the neighboring sequence environment. PhD-SNP classified the VHL variant p.Arg82Leu as deleterious. MutPred is a Random Forest-based classification method that utilizes several attributes related to protein structure, function, and evolution. MutPred also predicted that the variant p.Arg82Leu of VHL protein to be related to a pathogenic condition. PolyPhen2 utilizes a combination of sequence and structure based attributes for the description of an amino acid substitution, and the effect of mutation is predicted by a naive Bayesian classifier. The sequence-based features include Position Specific Independent Count (PSIC) scores and MSA properties, and position of mutation in relation to domain boundaries as defined by Pfam. PolyPhen2 predicted that the variant p.Arg82Leu probably damages the structure and function of VHL protein and gave a high PSIC score of 1. SNPs&GO is an SVM classifier based on mutation type and sequence environment information, sequence profiles taken from MSAs, predictions from the program Panther [47], and a function based log odds score describes information about protein function defined by Gene Ontology (GO) terms [48]. SNPs&GO predicted the VHL protein variant A82L as relevant to disease condition. All the six different tools used in this analysis predicted the variant p.Arg82Leu of VHL protein could lead to a disease condition. Disease causing mutations often reside in highly conserved positions in a protein sequence due to their functional importance. Further, SNPs that change amino acids are more likely to be associated with cancer susceptibility, a feature well documented in numerous comparative studies of human disease genes [49], [50], [51], [52]. We analyzed the evolutionary conservation of the amino acid change in the VHL protein by ConSurf to determine the degree of conservation of the mutant protein and notably, a high degree of conservation was identified which represents the evolutionary significance of the mutation (Figure 5). Further analysis was done using the project HOPE server for the p.Arg82Leu which showed deleterious effect by SIFT, I-Mutant3, PhD-SNP, MutPred, PolyPhen2 and SNPs&GO. HOPE combines structural information from a series of sources, including calculations on the 3D protein structure, sequence annotations in Uniprot and predictions from DAS-servers. Predicted properties of the native and mutant amino acid such as size, charge and hydrophobicity value are depicted in Table 2. Lastly, we subjected the p.Arg82Leu variant to molecular dynamic simulations to examine the extent of change in protein structure leading to a functionally disruptive effect.

**Table 1:** Prediction of deleterious effect in p.Arg82Leu variant using *in silico* prediction tools.

Prediction tools	Interpretation
SIFT	0.04
I-Mutant3	Disease
PhD-SNP	Disease
MutPred	Disease
PolyPhen2	1.00
SNPs&GO	Disease

**Table 2:** Evaluating the effect of the mutation by HOPE sever.

Amino acid properties	Native (Arginine)	Mutant (Leucine)	Effect of mutation
**Size**	Large	Small	Change in size leads to loss of external interactions
**Charge**	Positive	Neutral	Loss of charge leads to loss of interactions with other molecules
**Hydrophobicity**	Less	More	Increase in hydrophobicity leads to loss of hydrogen bonds made by the native amino acid residue to other monomers and affect the multimeric contacts. Arginine is involved in an ionic interaction, which might be important for multimerisation. This interaction is lost by this mutation.
**Contacts**	More	Lost	Mutant residue might be too small to make multimer contacts.
**Domains**	They are part of von Hippel-Lindau disease tumor suppressor, beta domain		The mutated residue is located on the surface of a domain with unknown function. The residue was not found to be in contact with other domains within the used structure. However, contact with other molecules is still possible and might be affected by this mutation

### Molecular Dynamics Structural Changes Analysis in Time Scale Level

**Figure 6:**
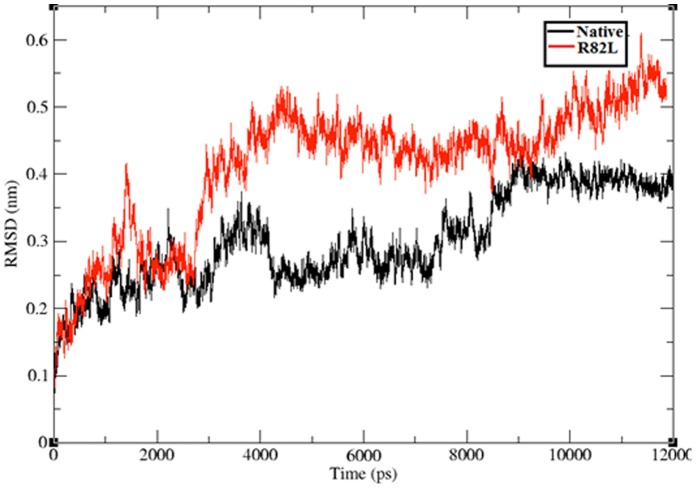
Backbone RMSDs shown as a function of time for native VHL protein (Black color) and mutant structure p.Arg82Leu (Red color). The ordinate is RMSD (nm), and the abscissa is time (ps).

Protein three dimensional (3D) structural information is an important feature for predicting the effects of deleterious variant and also provides information about the environment of the mutation. Proteins with mutations do not always have 3D structures that are analyzed and deposited in Protein data bank (PDB) necessitating the construction of 3D models by locating the mutation in 3D structures. This is a simple way of detecting the adverse effects of a mutation on a protein. Several researchers have utilized the structural information along with molecular dynamics to predict the impact of mutations in proteins and protein complexes [53], [54], [55], [56]. Indeed, a number of studies have shown good agreement between computational and experimental measurements of macromolecular dynamics [57], [58], [59], [60]. Thus, an atomic level look at the protein behavior using molecular dynamics simulations could help in better understanding the impact of the mutations on the protein structure, which in turn helped us in investigating how an amino acid variation can create a ripple effect throughout the protein structure and ultimately affect function. To understand the potential consequences of the p.Arg82Leu mutation on VHL protein structure and function, we carried out independent molecular dynamics simulations for each of the two following systems: native VHL protein and the p.Arg82Leu mutant to elucidate the atomic level changes with respect to the time scale. The time series of root mean square deviation (RMSD) are useful to visualize the spatial deviation of the structure during the simulation with respect to the energy minimized crystallographic conformation and it can explain the stability of molecules. We calculated the backbone RMSD for all the atoms from the initial structure and this was considered to be a central criterion to measure the convergence of the protein system concerned. The backbone RMSD were calculated for both the native and mutant structure from the respective trajectory files (Figure 6). The native structure obtained RMSD range from ∼0.2 nm to ∼0.4 nm whereas mutant structure p.Arg82Leu exhibited a deviation range from ∼0.2 to ∼0.55 nm. Difference in the deviation between the native and mutant RMSD trajectories form the starting conformation to their final states indicates the influence of residue substitutions on the dynamics and stability of the protein. VHL protein stability analysis results showed that the mutant protein obtained slightly high RMSD values when compared to the native protein. It is well established that higher deviation increases the stability of protein and high stability increases rigidity of the protein. Conformational changes are required for many protein functions [61], [62], [63], but the conformational flexibility and rigidity must be finely balanced [64]. Hence from the stability analysis of native and mutant proteins it was observed that mutant proteins obtained high rigidity due to the incorporation of deleterious variant at position 82 of VHL protein.

**Figure 7:**
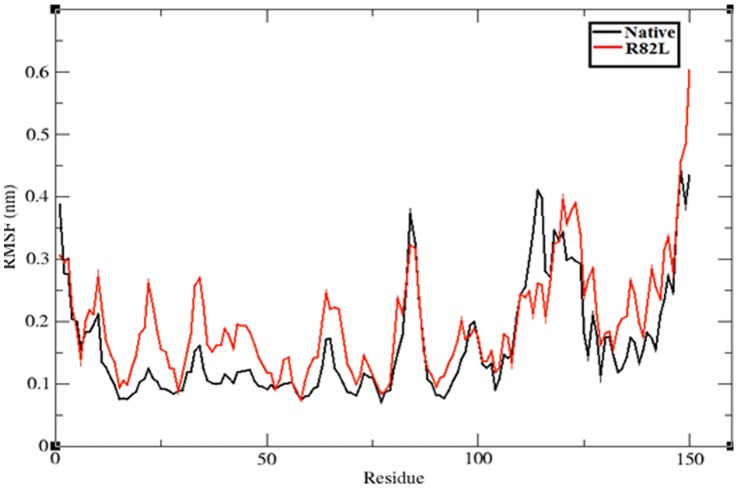
Central carbon alpha RMSF shown as a function of time for native VHL protein (Black color) and mutant structure p.Arg82Leu (Red color) respectively, at 300 K. The ordinate is RMSF (nm), and the abscissa is residues.

**Figure 8:**
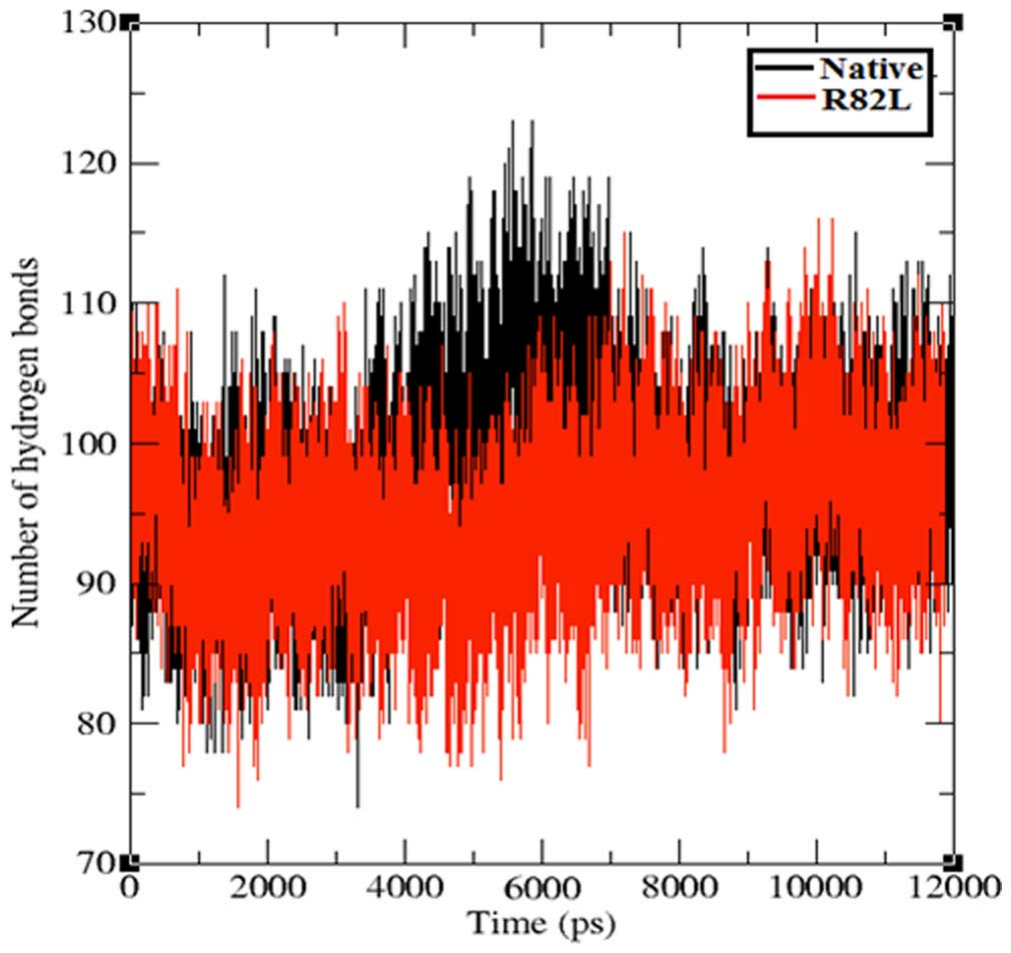
Average number of inter molecular hydrogen bonds in the native (Black color) and mutant protein p.Arg82Leu (Red color). The ordinate is number of hydrogen bond and the abscissa is time (ps).

To evaluate the structural flexibility of both native and mutant structures of VHL protein, we calculated the Root Mean Square Fluctuations (RMSFs) values from the 12 ns simulation trajectory data. The RMSFs values of native and mutant structures are shown in Figure 7. In the 12 ns simulation period, mutant structure residues ∼10 to ∼80 showed high fluctuation up to ∼0.25 nm, while native structure obtained high fluctuation from ∼0.15 nm to ∼0.2 nm in the same residue range of ∼10 to ∼80. In the remaining residue ranges from ∼80 to ∼150 alternative rise and fall in the RMSF were observed between the native and mutant structure. Overall the RMSFs of mutant structure were notably deviated from the native structure in the whole simulation period. Consistent with the RMSD analysis, change in the flexibility of mutant models reflected the deleterious effect of amino acid substitution in VHL protein. Hydrogen bonds and salt bridges are the important parameters in determining the stability of protein [65], [66]. nsSNPs can affect wild type protein structure and function by affecting hydrogen bond formations [53], [67]. Among the different kind of electrostatic forces, the intra molecular hydrogen bonds in protein act as a main contributor in maintaining the stability of protein structure. Incorporation of deleterious variant may change the possible electrostatic formation in molecule. Figure 8 depicts the number of hydrogen bonds formed in native and mutant structures of VHL protein. Native structure of VHL protein exhibits an average of ∼80 to ∼120 hydrogen bonds throughout the 12 ns simulation period and mutant structure obtained ∼80 to ∼110 hydrogen bonds in the whole simulation period. It was observed that significant numbers of hydrogen bonds were decreased in the mutant structures in comparison with the native structures. Hydrogen bond analysis results inferred that the occurrence of mutation destroyed the ability of hydrogen bond formation in p.Arg82Leu mutant structure. Hence, decrease in the number of hydrogen bonds was an indicator of a decreased stability in the mutant structure. The path traced by hydrogen bonds has been shown to be an important characteristic that may be used to infer the perturbation caused by a mutation [68].

**Figure 9:**
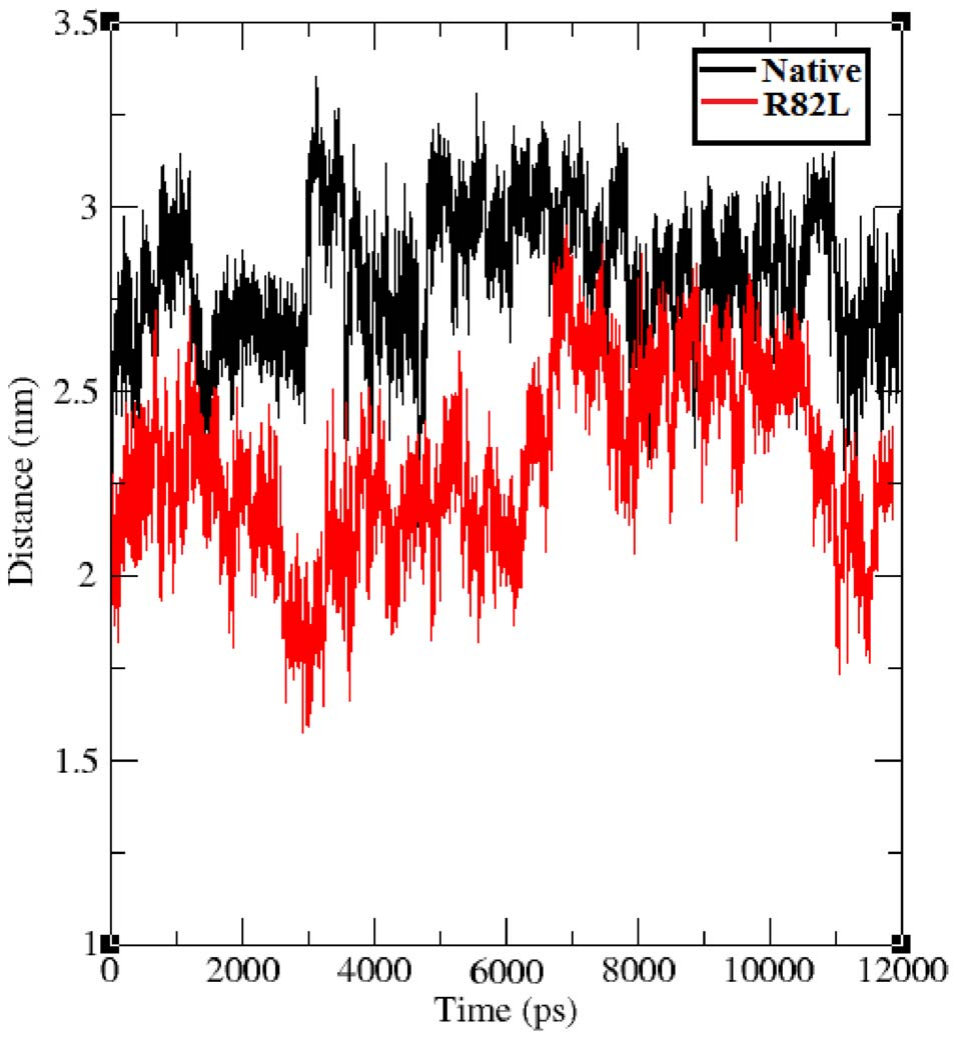
Distance between salt bridge forming residues in the native (Black color) and mutant protein p.Arg82Leu (Red color). The ordinate is distance (nm) and the abscissa is time (ps).

Interaction between positive and negative ions in proteins lead to salt bridges, which is an important stabilizing force and the presence of salt bridges, is an evidence for close proximity in the structure. Salt bridge distances of VHL protein in both native and mutant states were calculated from the 12 ns trajectory data and shown in Figure 9. Native structure of VHL protein had salt bridges and maintained different range of distances throughout the 12 ns simulation period. Native structure maintained salt bridge distance ranges from ∼2.5 nm to ∼3.5 nm and the mutant structure maintained the distance ranges from ∼1.7 nm to ∼2.7 nm. Comparing both the native and mutant structure salt bridge distance, p.Arg82Leu mutant structure obtained less distance between salt bridge forming residues. Decrease in the salt bridge distance in mutant indicates the substitution of amino acid makes the protein more compact than in the native state. It reflects that the modification occurred in the cationic side chains residues of mutant protein.

**Figure 10:**
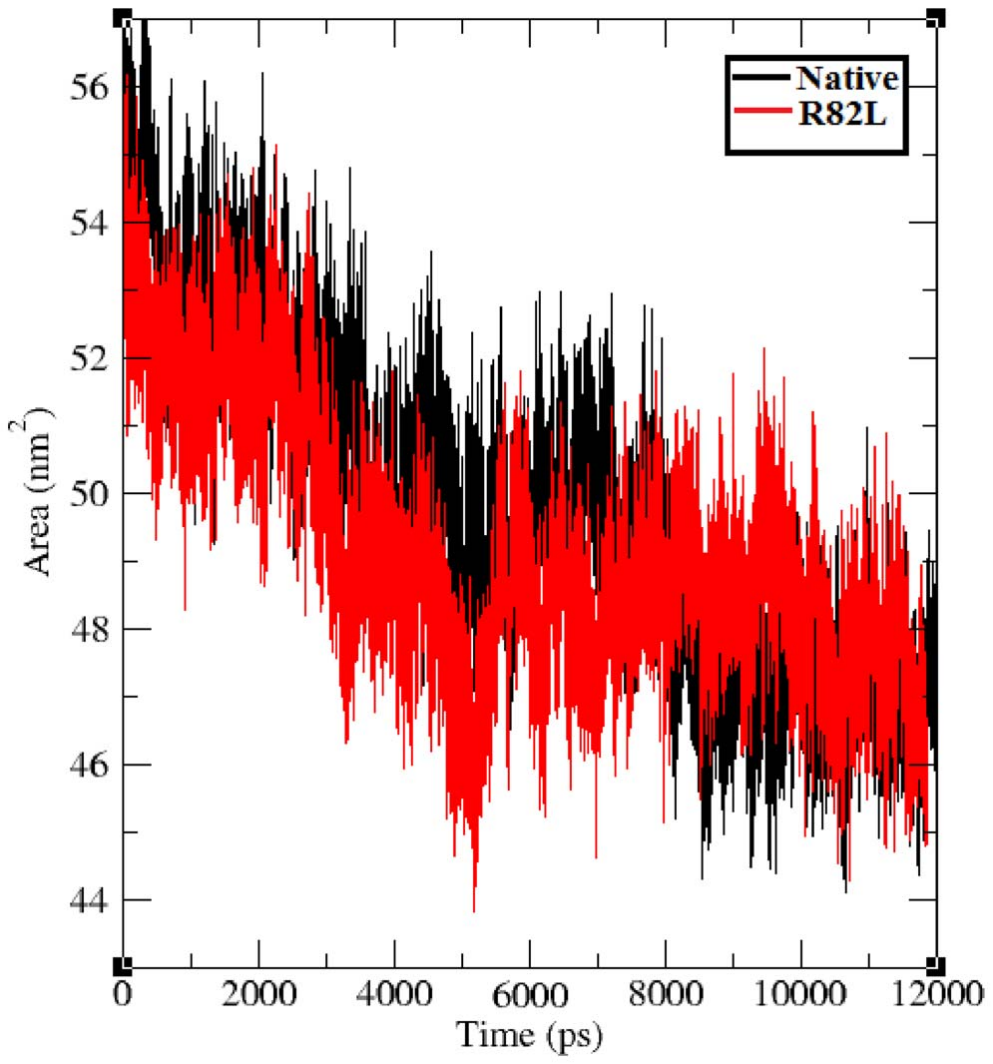
Solvent accessible surface of the native (Black color) and mutant protein p.Arg82Leu (Red color) over the entire simulation.

The solvent accessible surface area (SASA) of a biomolecule is that surface area traceable by the solvent molecule. These solvation effects can be calculated by explicit solvent models, such as MD simulations using a sphere of water molecules [69]. Solvent accessibility has been shown to be predominantly divided into buried and exposed region, indicating the least accessibility and high accessibility of the amino acid residues to the solvent [70]. SASA was calculated for both the native and mutant trajectory values of VHL protein. Figure 10 shows that the native VHL protein obtained SASA of ∼45 nm^2^ to ∼57 nm^2^ in the whole 12 ns simulation period. But all the mutant structure p.Arg82Leu obtained less SASA of ∼44 nm^2^ to ∼54 nm^2^. In comparison with the native protein, the mutant proteins obtained less SASA. Reduced SASA in the mutant proteins indicate that there may be change in the accessible area of amino acid residues from exposed to buried state vice versa. Lesser accessible area decreases the probability of regular interaction with biomolecules. Summing, all the *in silico* findings using different parameters such as RMSD, RMSF, salt bridge, H-bonds and SASA applied in molecular dynamic study, the variant p.Arg82Leu was found to alter the structure of the protein and to have a pathogenic potential.

### Conclusions

All the three patients with the p.Arg82Leu mutation have been on long follow-up, with the index case being followed up for ∼40 years. Interestingly, all these patients have remained asymptomatic despite the prediction tools used in this study predicting the mutation to have a pathogenic potential. Further, analyzing the conformational changes of amino acid residue within VHL protein has helped to identify significant structural changes. Whether these patients will eventually develop symptoms or remain asymptomatic through their lifetime, indicating that the risks associated with carrying the p.Arg82Leu mutation is minimal, is a feature that can only be figured with a passage of time.
